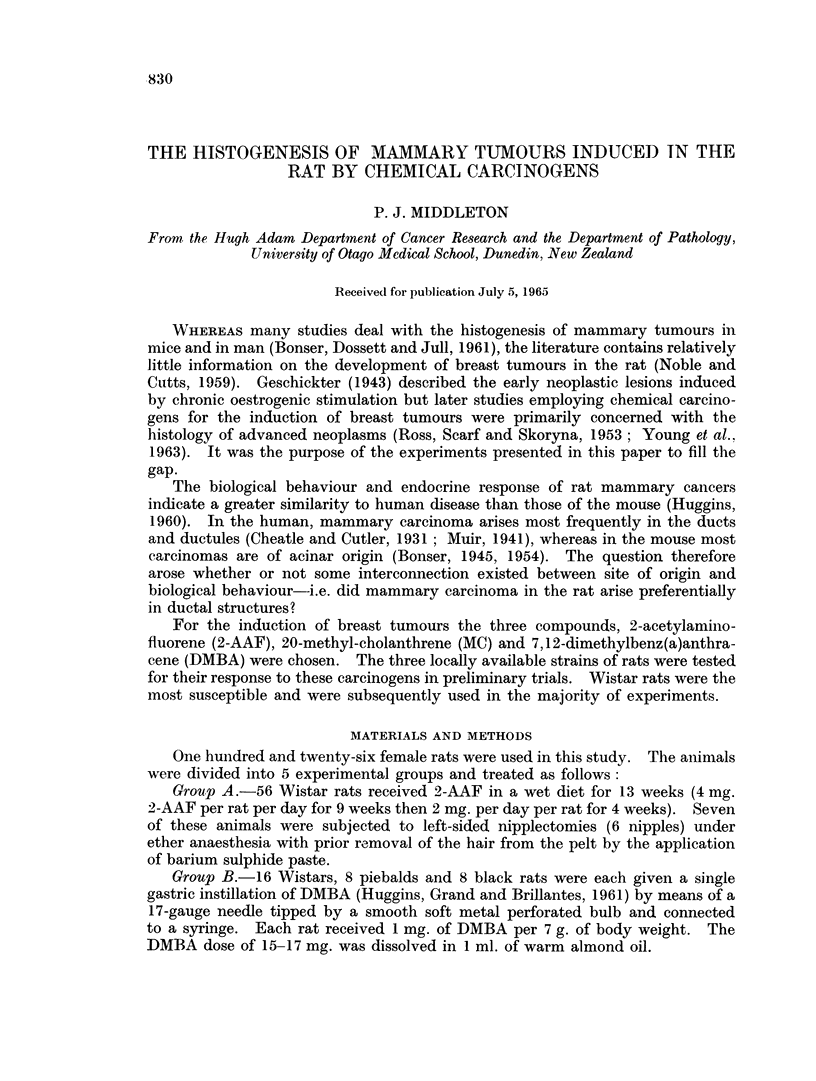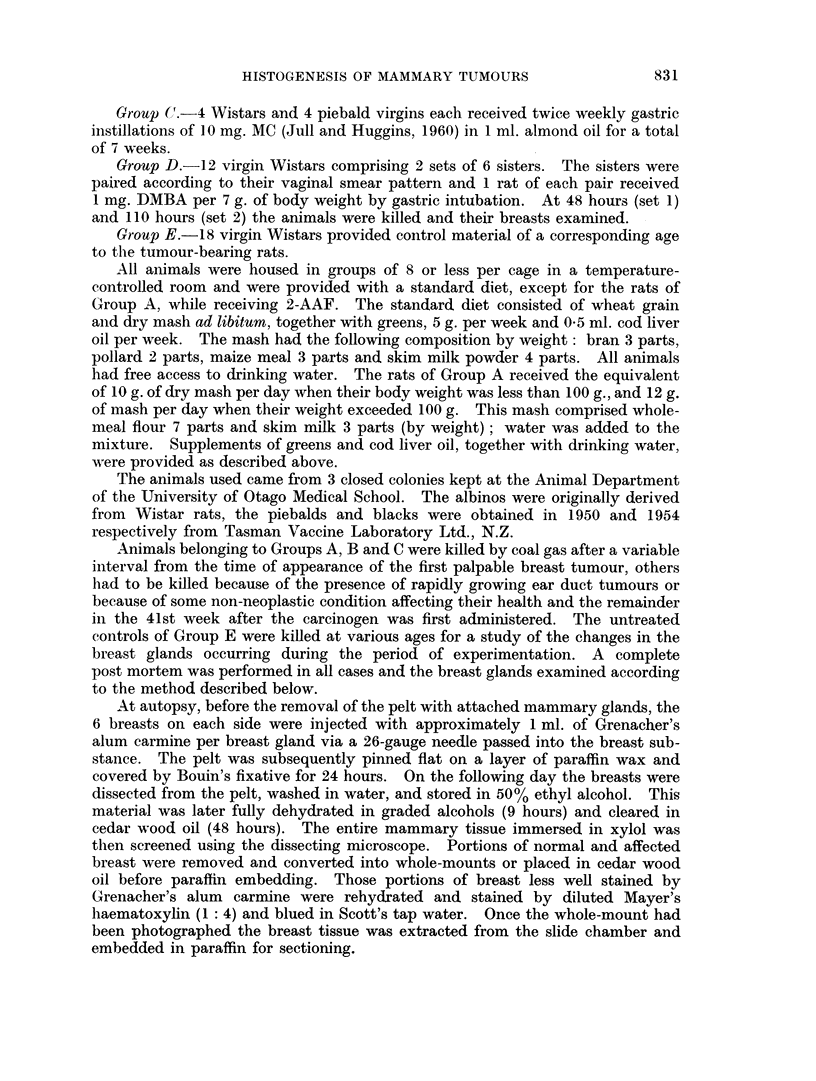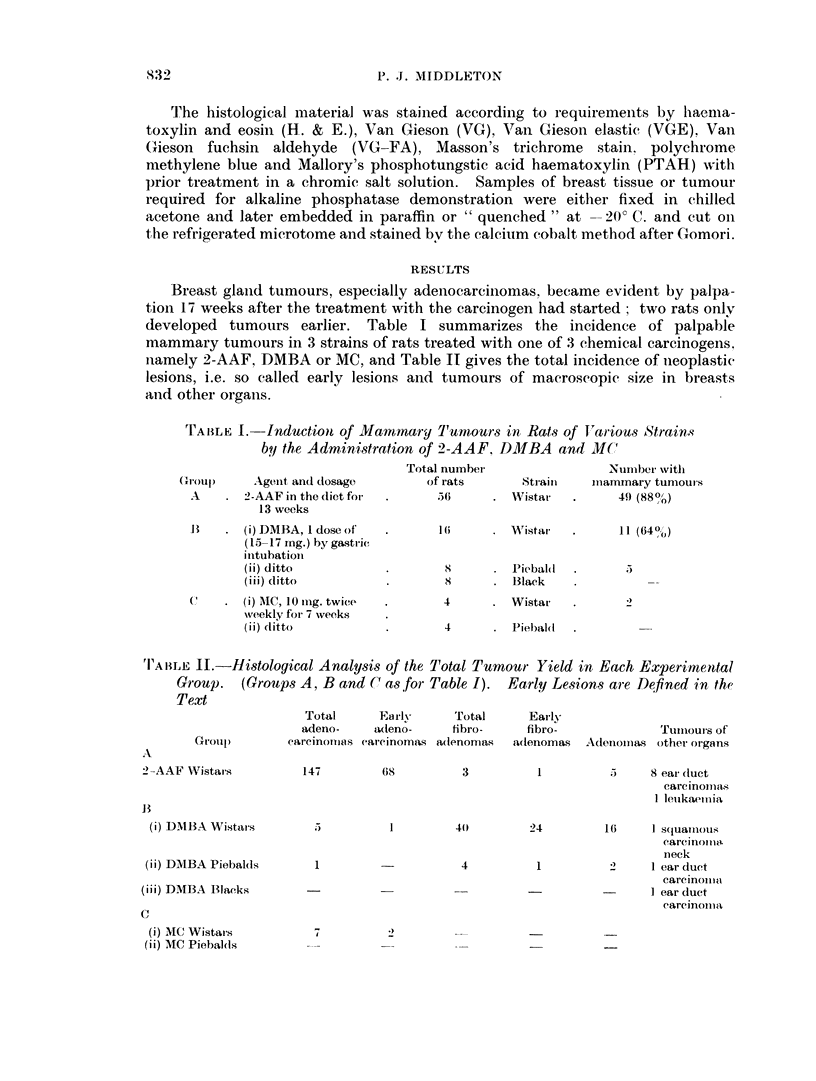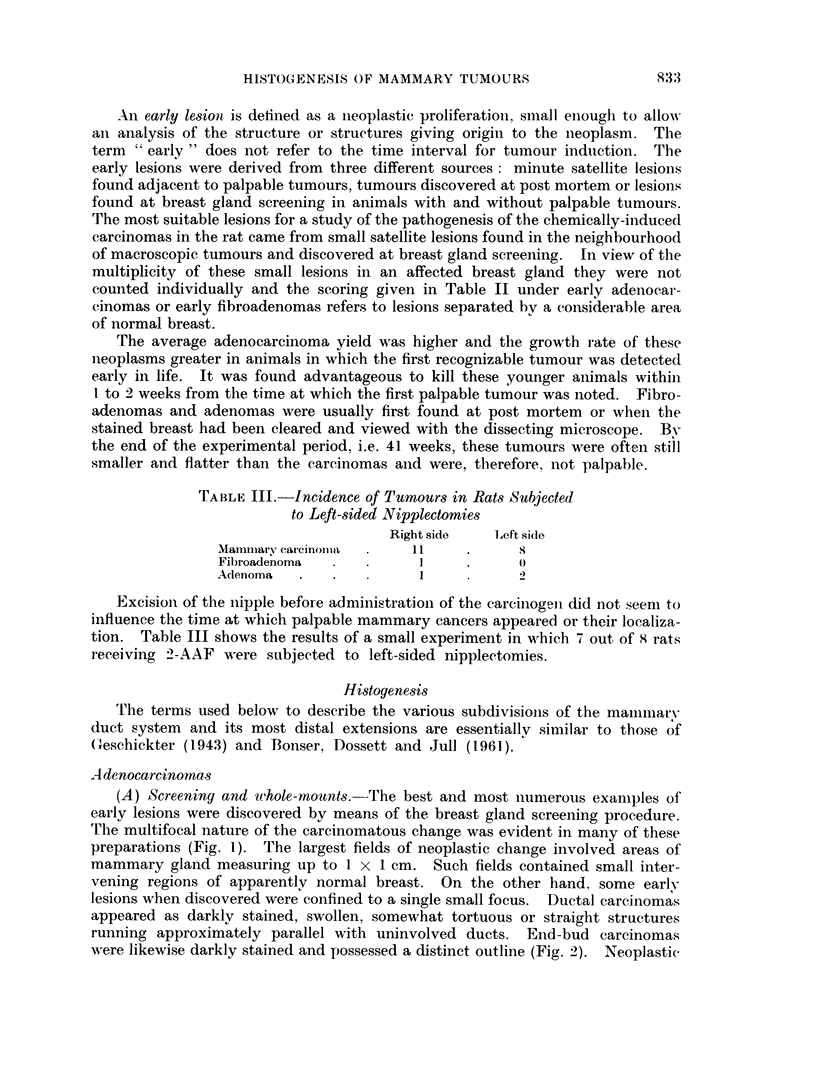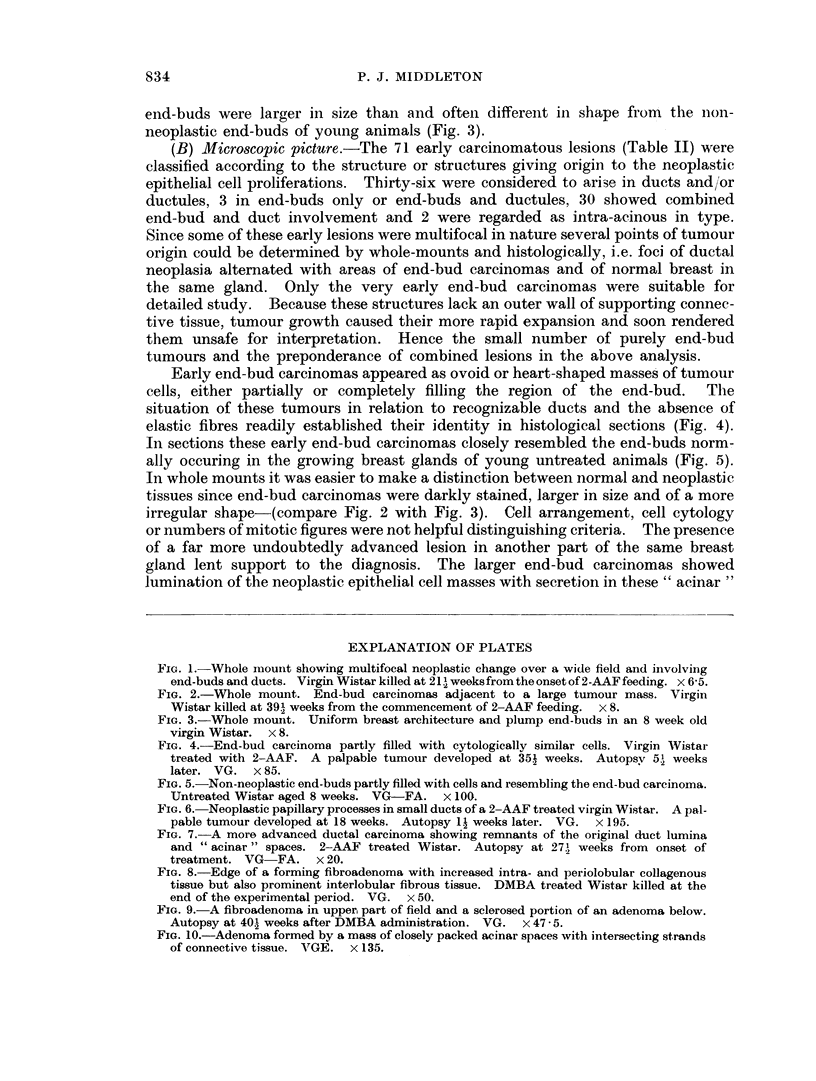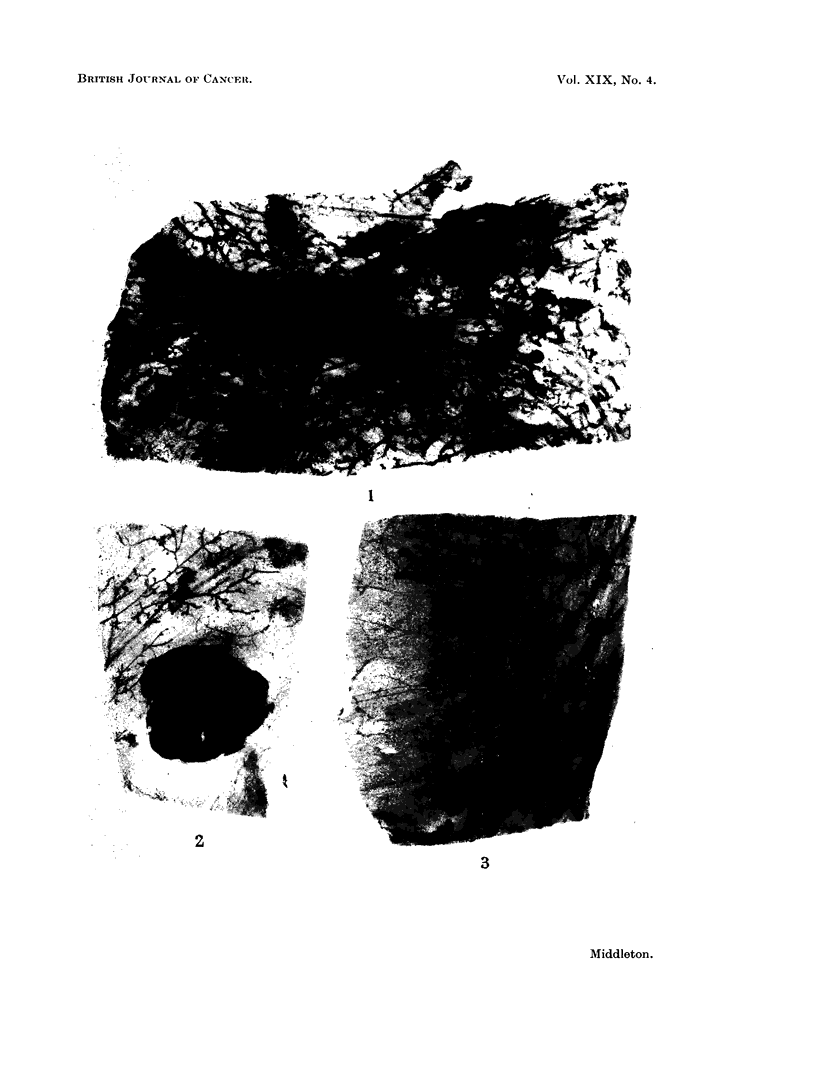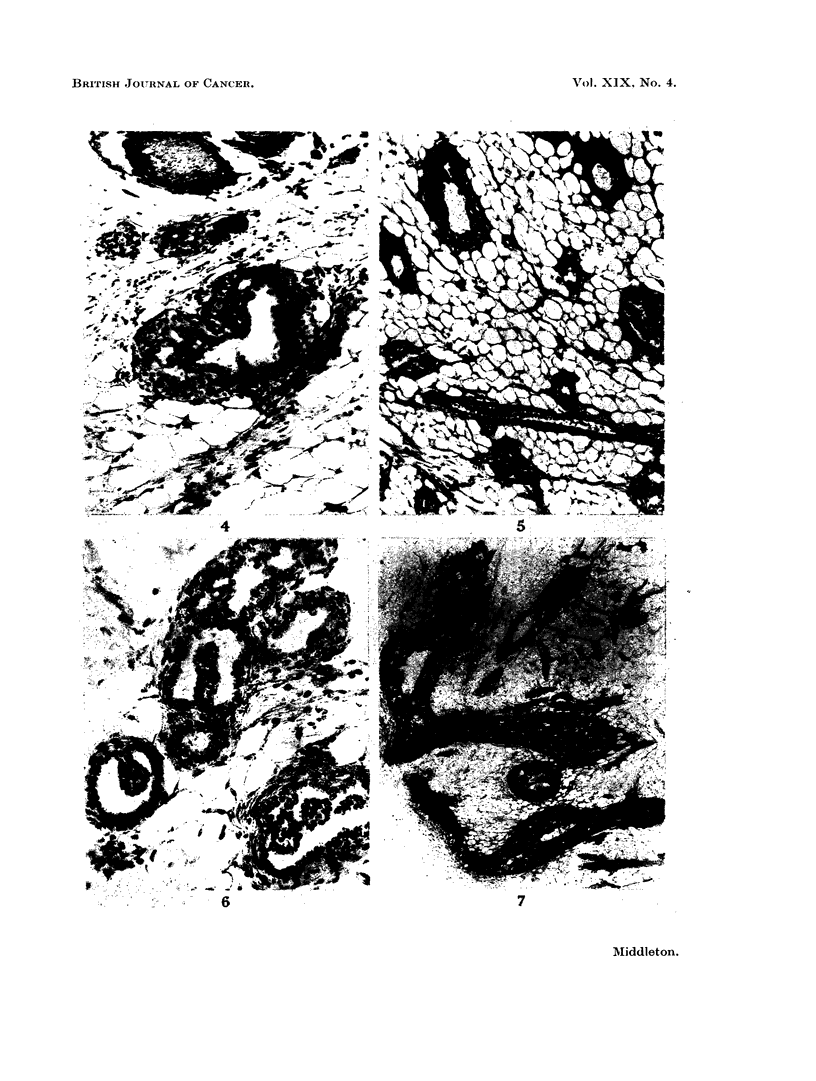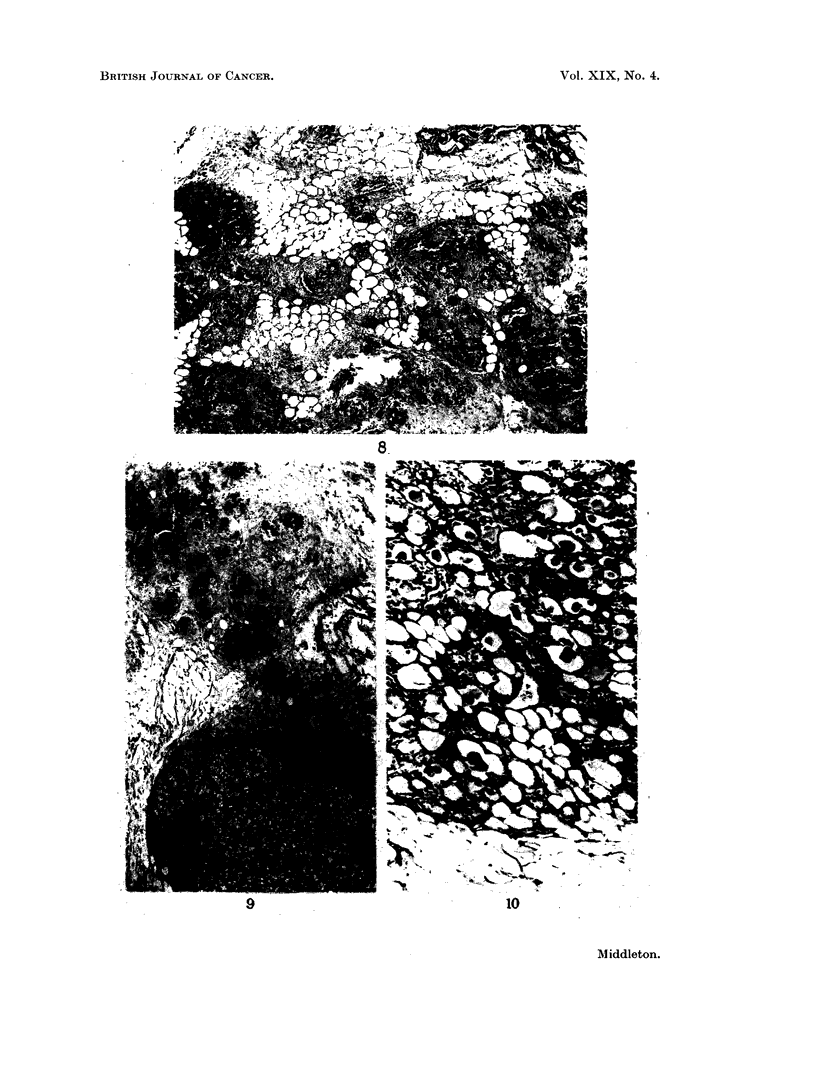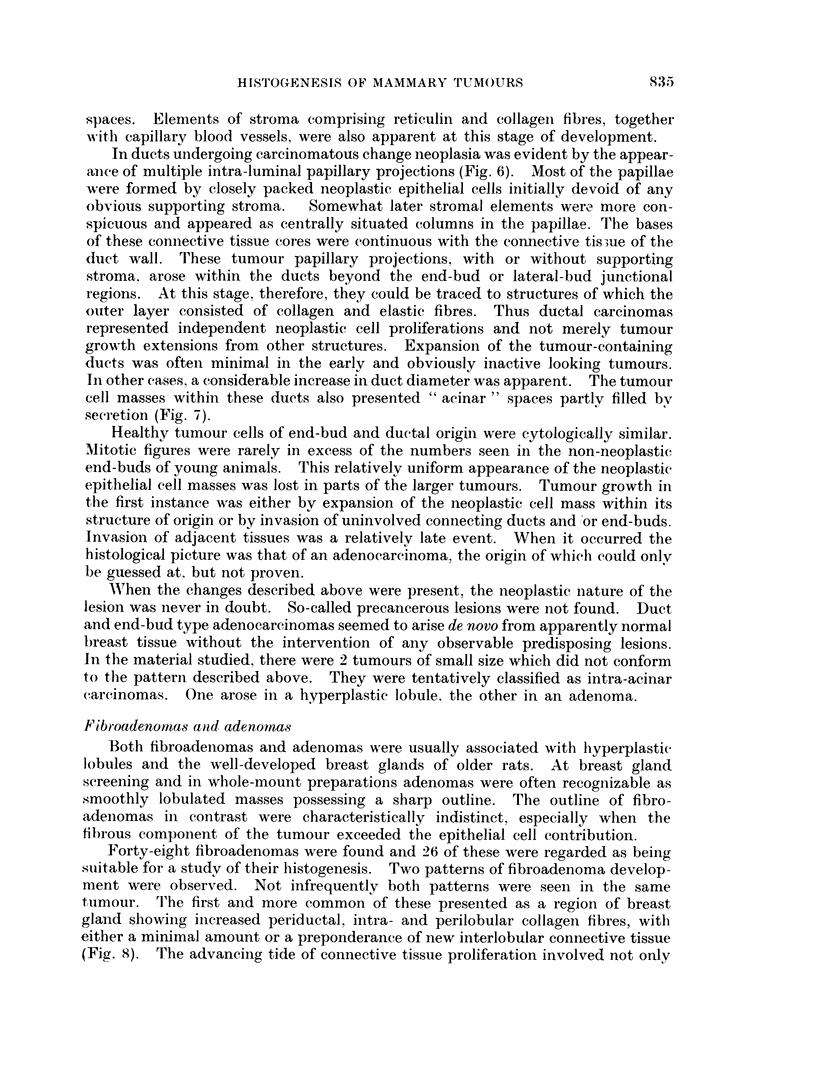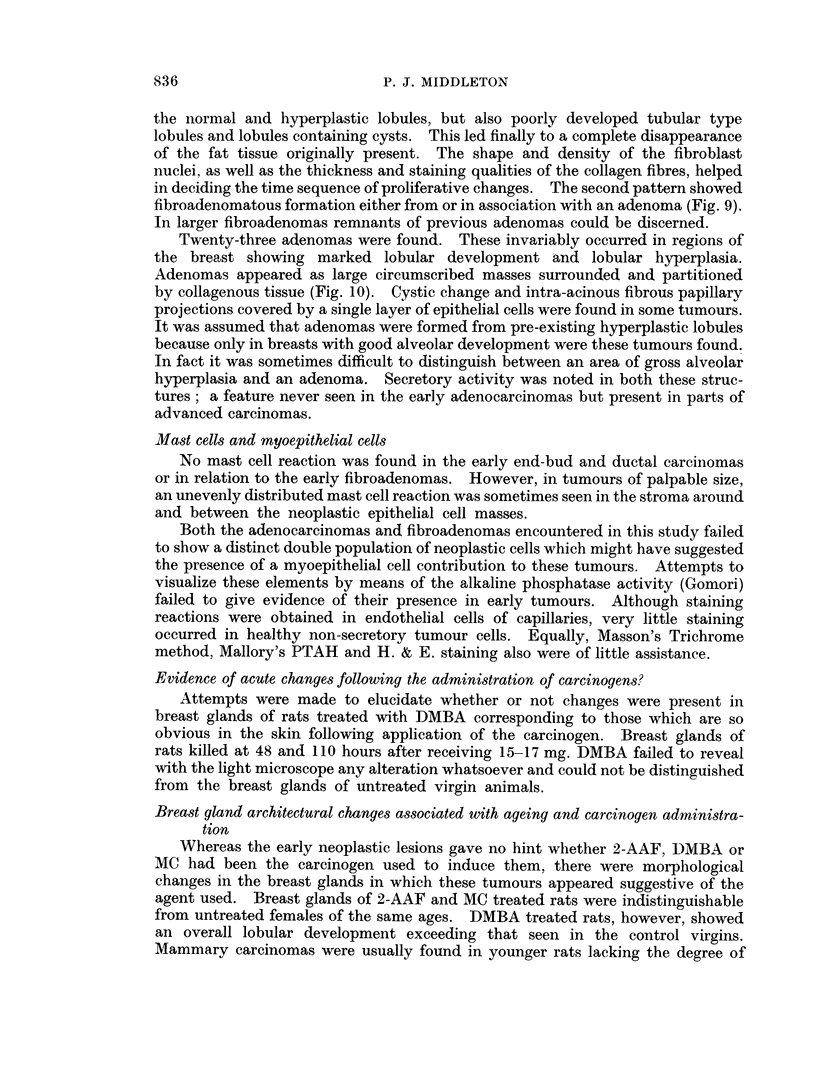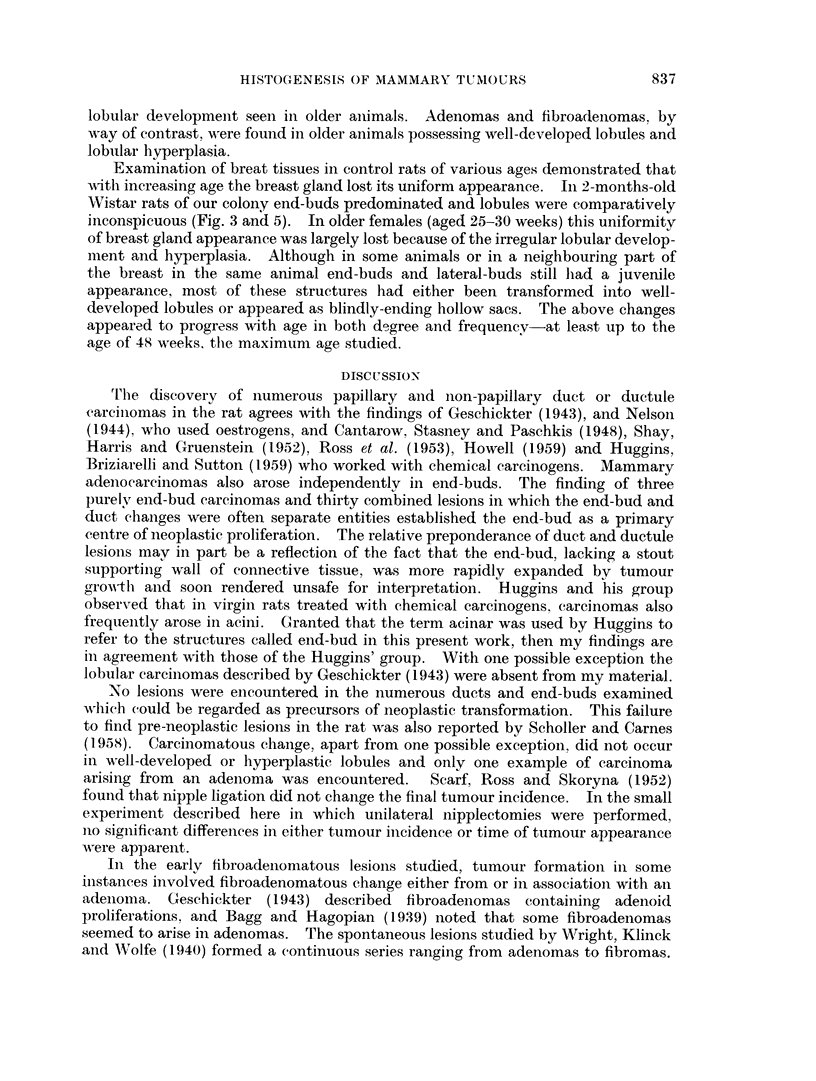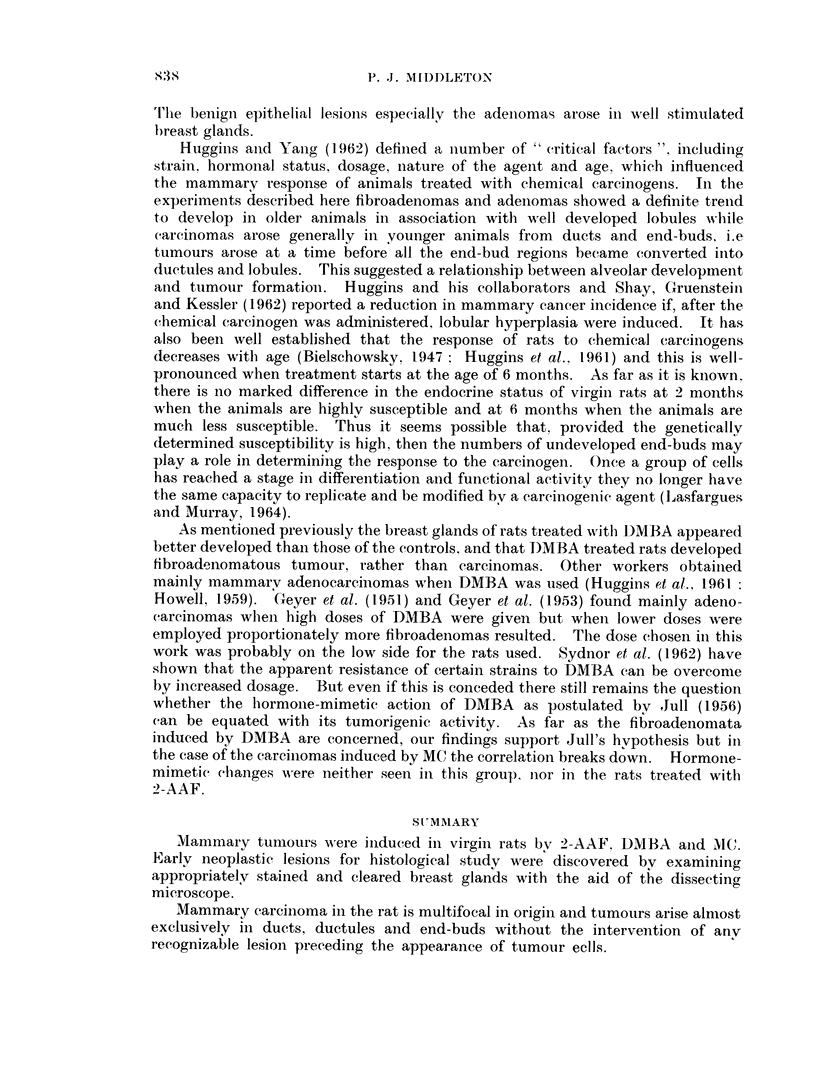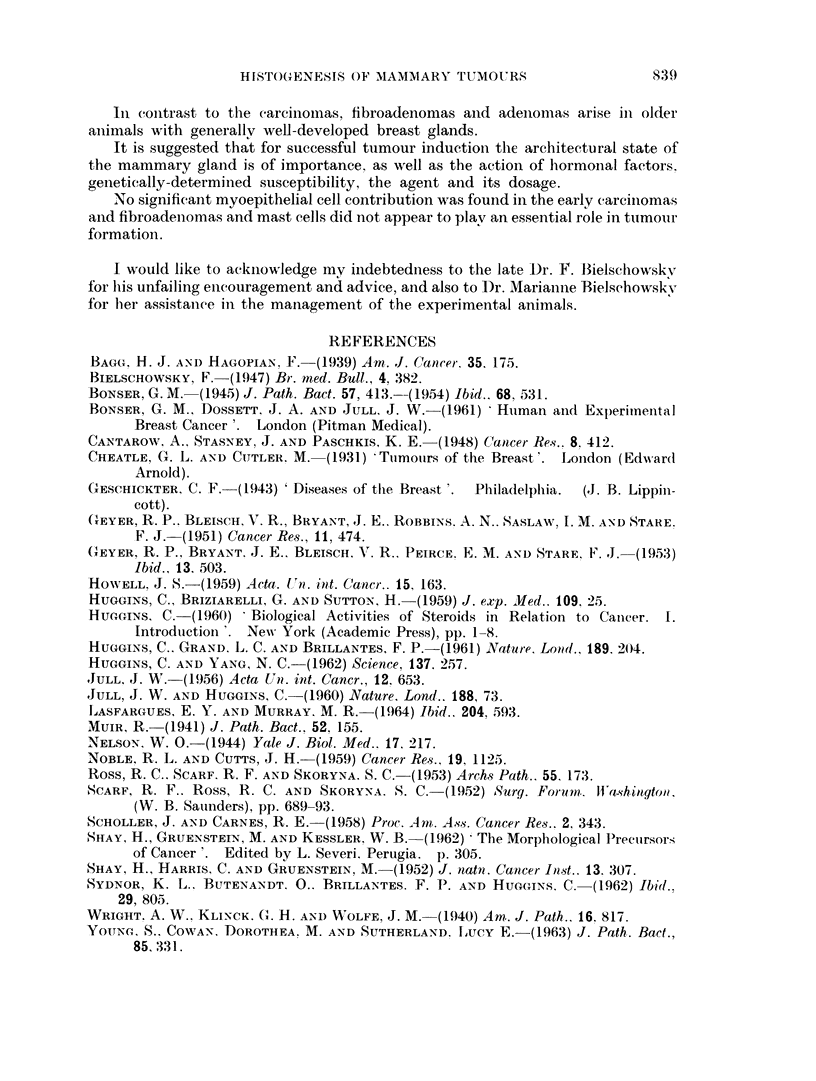# The histogenesis of mammary tumours induced in the rat by chemical carcinogens.

**DOI:** 10.1038/bjc.1965.96

**Published:** 1965-12

**Authors:** P. J. Middleton

## Abstract

**Images:**


### THE HISTOGENESIS OF MAMMARY TUMOURS INDUCED IN THE RAT BY CHEMICAL CARCINOGENS

P. J. MIDDLETON

*From the Hugh Adam Department of Cancer Research and the Department of Pathology, University of Otago Medical School, Dunedin, New Zealand*

Received for publication July 5, 1965

WHEREAS many studies deal with the histogenesis of mammary tumours in mice and in man (Bonser, Dossett and Jull, 1961), the literature contains relatively little information on the development of breast tumours in the rat (Noble and Cutts, 1959). Geschickter (1943) described the early neoplastic lesions induced by chronic oestrogenic stimulation but later studies employing chemical carcinogens for the induction of breast tumours were primarily concerned with the histology of advanced neoplasms (Ross, Scarf and Skoryna, 1953 ; Young *et al.*, 1963). It was the purpose of the experiments presented in this paper to fill the gap.

The biological behaviour and endocrine response of rat mammary cancers indicate a greater similarity to human disease than those of the mouse (Huggins, 1960). In the human, mammary carcinoma arises most frequently in the ducts and ductules (Cheatle and Cutler, 1931 ; Muir, 1941), whereas in the mouse most carcinomas are of acinar origin (Bonser, 1945, 1954). The question therefore arose whether or not some interconnection existed between site of origin and biological behaviour—i.e. did mammary carcinoma in the rat arise preferentially in ductal structures?

For the induction of breast tumours the three compounds, 2-acetylamino-fluorene (2-AAF), 20-methyl-cholanthrene (MC) and 7,12-dimethylbenz(a)anthracene (DMBA) were chosen. The three locally available strains of rats were tested for their response to these carcinogens in preliminary trials. Wistar rats were the most susceptible and were subsequently used in the majority of experiments.

### MATERIALS AND METHODS

One hundred and twenty-six female rats were used in this study. The animals were divided into 5 experimental groups and treated as follows :

*Group A.*—56 Wistar rats received 2-AAF in a wet diet for 13 weeks (4 mg. 2-AAF per rat per day for 9 weeks then 2 mg. per day per rat for 4 weeks). Seven of these animals were subjected to left-sided nipplectomies (6 nipples) under ether anaesthesia with prior removal of the hair from the pelt by the application of barium sulphide paste.

*Group B.*—16 Wistars, 8 piebalds and 8 black rats were each given a single gastric instillation of DMBA (Huggins, Grand and Brillantes, 1961) by means of a 17-gauge needle tipped by a smooth soft metal perforated bulb and connected to a syringe. Each rat received 1 mg. of DMBA per 7 g. of body weight. The DMBA dose of 15–17 mg. was dissolved in 1 ml. of warm almond oil.

*Group C.*—4 Wistars and 4 piebald virgins each received twice weekly gastric instillations of 10 mg. MC (Jull and Huggins, 1960) in 1 ml. almond oil for a total of 7 weeks.

*Group D.*—12 virgin Wistars comprising 2 sets of 6 sisters. The sisters were paired according to their vaginal smear pattern and 1 rat of each pair received 1 mg. DMBA per 7 g. of body weight by gastric intubation. At 48 hours (set 1) and 110 hours (set 2) the animals were killed and their breasts examined.

*Group E.*—18 virgin Wistars provided control material of a corresponding age to the tumour-bearing rats.

All animals were housed in groups of 8 or less per cage in a temperature-controlled room and were provided with a standard diet, except for the rats of Group A, while receiving 2-AAF. The standard diet consisted of wheat grain and dry mash *ad libitum*, together with greens, 5 g. per week and 0·5 ml. cod liver oil per week. The mash had the following composition by weight : bran 3 parts, pollard 2 parts, maize meal 3 parts and skim milk powder 4 parts. All animals had free access to drinking water. The rats of Group A received the equivalent of 10 g. of dry mash per day when their body weight was less than 100 g., and 12 g. of mash per day when their weight exceeded 100 g. This mash comprised wholemeal flour 7 parts and skim milk 3 parts (by weight) ; water was added to the mixture. Supplements of greens and cod liver oil, together with drinking water, were provided as described above.

The animals used came from 3 closed colonies kept at the Animal Department of the University of Otago Medical School. The albinos were originally derived from Wistar rats, the piebalds and blacks were obtained in 1950 and 1954 respectively from Tasman Vaccine Laboratory Ltd., N.Z.

Animals belonging to Groups A, B and C were killed by coal gas after a variable interval from the time of appearance of the first palpable breast tumour, others had to be killed because of the presence of rapidly growing ear duct tumours or because of some non-neoplastic condition affecting their health and the remainder in the 41st week after the carcinogen was first administered. The untreated controls of Group E were killed at various ages for a study of the changes in the breast glands occurring during the period of experimentation. A complete post mortem was performed in all cases and the breast glands examined according to the method described below.

At autopsy, before the removal of the pelt with attached mammary glands, the 6 breasts on each side were injected with approximately 1 ml. of Grenacher's alum carmine per breast gland via a 26-gauge needle passed into the breast substance. The pelt was subsequently pinned flat on a layer of paraffin wax and covered by Bouin's fixative for 24 hours. On the following day the breasts were dissected from the pelt, washed in water, and stored in 50% ethyl alcohol. This material was later fully dehydrated in graded alcohols (9 hours) and cleared in cedar wood oil (48 hours). The entire mammary tissue immersed in xylol was then screened using the dissecting microscope. Portions of normal and affected breast were removed and converted into whole-mounts or placed in cedar wood oil before paraffin embedding. Those portions of breast less well stained by Grenacher's alum carmine were rehydrated and stained by diluted Mayer's haematoxylin (1 : 4) and blued in Scott's tap water. Once the whole-mount had been photographed the breast tissue was extracted from the slide chamber and embedded in paraffin for sectioning.

The histological material was stained according to requirements by haematoxylin and eosin (H. & E.), Van Gieson (VG), Van Gieson elastic (VGE), Van Gieson fuchsin aldehyde (VG–FA), Masson's trichrome stain, polychrome methylene blue and Mallory's phosphotungstic acid haematoxylin (PTAH) with prior treatment in a chromic salt solution. Samples of breast tissue or tumour required for alkaline phosphatase demonstration were either fixed in chilled acetone and later embedded in paraffin or " quenched " at $-20°$ C. and cut on the refrigerated microtome and stained by the calcium cobalt method after Gomori.

### RESULTS

Breast gland tumours, especially adenocarcinomas, became evident by palpation 17 weeks after the treatment with the carcinogen had started ; two rats only developed tumours earlier. Table I summarizes the incidence of palpable mammary tumours in 3 strains of rats treated with one of 3 chemical carcinogens, namely 2-AAF, DMBA or MC, and Table II gives the total incidence of neoplastic lesions, i.e. so called early lesions and tumours of macroscopic size in breasts and other organs.

TABLE I.—*Induction of Mammary Tumours in Rats of Various Strains by the Administration of 2-AAF, DMBA and MC*

| Group | Agent and dosage | Total number of rats | Strain | Number with mammary tumours |
|---|---|---|---|---|
| A | 2-AAF in the diet for 13 weeks | 56 | Wistar | 49 (88%) |
| B | (i) DMBA, 1 dose of (15–17 mg.) by gastric intubation | 16 | Wistar | 11 (64%) |
| | (ii) ditto | 8 | Piebald | 5 |
| | (iii) ditto | 8 | Black | — |
| C | (i) MC, 10 mg. twice weekly for 7 weeks | 4 | Wistar | 2 |
| | (ii) ditto | 4 | Piebald | — |

TABLE II.—*Histological Analysis of the Total Tumour Yield in Each Experimental Group.* (*Groups A, B and C as for Table I*). *Early Lesions are Defined in the Text*

| Group | Total adeno-carcinomas | Early adeno-carcinomas | Total fibro-adenomas | Early fibro-adenomas | Adenomas | Tumours of other organs |
|---|---|---|---|---|---|---|
| A | | | | | | |
| 2-AAF Wistars | 147 | 68 | 3 | 1 | 5 | 8 ear duct carcinomas<br>1 leukaemia |
| B | | | | | | |
| (i) DMBA Wistars | 5 | 1 | 40 | 24 | 16 | 1 squamous carcinoma neck |
| (ii) DMBA Piebalds | 1 | — | 4 | 1 | 2 | 1 ear duct carcinoma |
| (iii) DMBA Blacks | — | — | — | — | — | 1 ear duct carcinoma |
| C | | | | | | |
| (i) MC Wistars | 7 | 2 | — | — | — | |
| (ii) MC Piebalds | — | — | — | — | — | |

An *early lesion* is defined as a neoplastic proliferation, small enough to allow an analysis of the structure or structures giving origin to the neoplasm. The term " early " does not refer to the time interval for tumour induction. The early lesions were derived from three different sources : minute satellite lesions found adjacent to palpable tumours, tumours discovered at post mortem or lesions found at breast gland screening in animals with and without palpable tumours. The most suitable lesions for a study of the pathogenesis of the chemically-induced carcinomas in the rat came from small satellite lesions found in the neighbourhood of macroscopic tumours and discovered at breast gland screening. In view of the multiplicity of these small lesions in an affected breast gland they were not counted individually and the scoring given in Table II under early adenocarcinomas or early fibroadenomas refers to lesions separated by a considerable area of normal breast.

The average adenocarcinoma yield was higher and the growth rate of these neoplasms greater in animals in which the first recognizable tumour was detected early in life. It was found advantageous to kill these younger animals within 1 to 2 weeks from the time at which the first palpable tumour was noted. Fibroadenomas and adenomas were usually first found at post mortem or when the stained breast had been cleared and viewed with the dissecting microscope. By the end of the experimental period, i.e. 41 weeks, these tumours were often still smaller and flatter than the carcinomas and were, therefore, not palpable.

TABLE III.—*Incidence of Tumours in Rats Subjected to Left-sided Nipplectomies*

| | Right side | Left side |
|---|---|---|
| Mammary carcinoma | 11 | 8 |
| Fibroadenoma | 1 | 0 |
| Adenoma | 1 | 2 |

Excision of the nipple before administration of the carcinogen did not seem to influence the time at which palpable mammary cancers appeared or their localization. Table III shows the results of a small experiment in which 7 out of 8 rats receiving 2-AAF were subjected to left-sided nipplectomies.

### *Histogenesis*

The terms used below to describe the various subdivisions of the mammary duct system and its most distal extensions are essentially similar to those of Geschickter (1943) and Bonser, Dossett and Jull (1961).

#### *Adenocarcinomas*

(*A*) *Screening and whole-mounts.*—The best and most numerous examples of early lesions were discovered by means of the breast gland screening procedure. The multifocal nature of the carcinomatous change was evident in many of these preparations (Fig. 1). The largest fields of neoplastic change involved areas of mammary gland measuring up to 1 × 1 cm. Such fields contained small intervening regions of apparently normal breast. On the other hand, some early lesions when discovered were confined to a single small focus. Ductal carcinomas appeared as darkly stained, swollen, somewhat tortuous or straight structures running approximately parallel with uninvolved ducts. End-bud carcinomas were likewise darkly stained and possessed a distinct outline (Fig. 2). Neoplastic end-buds were larger in size than and often different in shape from the non-neoplastic end-buds of young animals (Fig. 3).

(*B*) *Microscopic picture.*—The 71 early carcinomatous lesions (Table II) were classified according to the structure or structures giving origin to the neoplastic epithelial cell proliferations. Thirty-six were considered to arise in ducts and/or ductules, 3 in end-buds only or end-buds and ductules, 30 showed combined end-bud and duct involvement and 2 were regarded as intra-acinous in type. Since some of these early lesions were multifocal in nature several points of tumour origin could be determined by whole-mounts and histologically, i.e. foci of ductal neoplasia alternated with areas of end-bud carcinomas and of normal breast in the same gland. Only the very early end-bud carcinomas were suitable for detailed study. Because these structures lack an outer wall of supporting connective tissue, tumour growth caused their more rapid expansion and soon rendered them unsafe for interpretation. Hence the small number of purely end-bud tumours and the preponderance of combined lesions in the above analysis.

Early end-bud carcinomas appeared as ovoid or heart-shaped masses of tumour cells, either partially or completely filling the region of the end-bud. The situation of these tumours in relation to recognizable ducts and the absence of elastic fibres readily established their identity in histological sections (Fig. 4). In sections these early end-bud carcinomas closely resembled the end-buds normally occuring in the growing breast glands of young untreated animals (Fig. 5). In whole mounts it was easier to make a distinction between normal and neoplastic tissues since end-bud carcinomas were darkly stained, larger in size and of a more irregular shape—(compare Fig. 2 with Fig. 3). Cell arrangement, cell cytology or numbers of mitotic figures were not helpful distinguishing criteria. The presence of a far more undoubtedly advanced lesion in another part of the same breast gland lent support to the diagnosis. The larger end-bud carcinomas showed lumination of the neoplastic epithelial cell masses with secretion in these " acinar "

EXPLANATION OF PLATES

FIG. 1.—Whole mount showing multifocal neoplastic change over a wide field and involving end-buds and ducts. Virgin Wistar killed at 21½ weeks from the onset of 2-AAF feeding. ×6·5.

FIG. 2.—Whole mount. End-bud carcinomas adjacent to a large tumour mass. Virgin Wistar killed at 39½ weeks from the commencement of 2–AAF feeding. ×8.

FIG. 3.—Whole mount. Uniform breast architecture and plump end-buds in an 8 week old virgin Wistar. ×8.

FIG. 4.—End-bud carcinoma partly filled with cytologically similar cells. Virgin Wistar treated with 2–AAF. A palpable tumour developed at 35½ weeks. Autopsy 5½ weeks later. VG. ×85.

FIG. 5.—Non-neoplastic end-buds partly filled with cells and resembling the end-bud carcinoma. Untreated Wistar aged 8 weeks. VG—FA. ×100.

FIG. 6.—Neoplastic papillary processes in small ducts of a 2–AAF treated virgin Wistar. A palpable tumour developed at 18 weeks. Autopsy 1½ weeks later. VG. ×195.

FIG. 7.—A more advanced ductal carcinoma showing remnants of the original duct lumina and " acinar " spaces. 2–AAF treated Wistar. Autopsy at 27½ weeks from onset of treatment. VG—FA. ×20.

FIG. 8.—Edge of a forming fibroadenoma with increased intra- and periolobular collagenous tissue but also prominent interlobular fibrous tissue. DMBA treated Wistar killed at the end of the experimental period. VG. ×50.

FIG. 9.—A fibroadenoma in upper part of field and a sclerosed portion of an adenoma below. Autopsy at 40½ weeks after DMBA administration. VG. ×47·5.

FIG. 10.—Adenoma formed by a mass of closely packed acinar spaces with intersecting strands of connective tissue. VGE. ×135.

1

2

3

4

5

6

7

Middleton.

8

9

10

spaces. Elements of stroma comprising reticulin and collagen fibres, together with capillary blood vessels, were also apparent at this stage of development.

In ducts undergoing carcinomatous change neoplasia was evident by the appearance of multiple intra-luminal papillary projections (Fig. 6). Most of the papillae were formed by closely packed neoplastic epithelial cells initially devoid of any obvious supporting stroma. Somewhat later stromal elements were more conspicuous and appeared as centrally situated columns in the papillae. The bases of these connective tissue cores were continuous with the connective tissue of the duct wall. These tumour papillary projections, with or without supporting stroma, arose within the ducts beyond the end-bud or lateral-bud junctional regions. At this stage, therefore, they could be traced to structures of which the outer layer consisted of collagen and elastic fibres. Thus ductal carcinomas represented independent neoplastic cell proliferations and not merely tumour growth extensions from other structures. Expansion of the tumour-containing ducts was often minimal in the early and obviously inactive looking tumours. In other cases, a considerable increase in duct diameter was apparent. The tumour cell masses within these ducts also presented " acinar " spaces partly filled by secretion (Fig. 7).

Healthy tumour cells of end-bud and ductal origin were cytologically similar. Mitotic figures were rarely in excess of the numbers seen in the non-neoplastic end-buds of young animals. This relatively uniform appearance of the neoplastic epithelial cell masses was lost in parts of the larger tumours. Tumour growth in the first instance was either by expansion of the neoplastic cell mass within its structure of origin or by invasion of uninvolved connecting ducts and/or end-buds. Invasion of adjacent tissues was a relatively late event. When it occurred the histological picture was that of an adenocarcinoma, the origin of which could only be guessed at, but not proven.

When the changes described above were present, the neoplastic nature of the lesion was never in doubt. So-called precancerous lesions were not found. Duct and end-bud type adenocarcinomas seemed to arise *de novo* from apparently normal breast tissue without the intervention of any observable predisposing lesions. In the material studied, there were 2 tumours of small size which did not conform to the pattern described above. They were tentatively classified as intra-acinar carcinomas. One arose in a hyperplastic lobule, the other in an adenoma.

*Fibroadenomas and adenomas*

Both fibroadenomas and adenomas were usually associated with hyperplastic lobules and the well-developed breast glands of older rats. At breast gland screening and in whole-mount preparations adenomas were often recognizable as smoothly lobulated masses possessing a sharp outline. The outline of fibroadenomas in contrast were characteristically indistinct, especially when the fibrous component of the tumour exceeded the epithelial cell contribution.

Forty-eight fibroadenomas were found and 26 of these were regarded as being suitable for a study of their histogenesis. Two patterns of fibroadenoma development were observed. Not infrequently both patterns were seen in the same tumour. The first and more common of these presented as a region of breast gland showing increased periductal, intra- and perilobular collagen fibres, with either a minimal amount or a preponderance of new interlobular connective tissue (Fig. 8). The advancing tide of connective tissue proliferation involved not only the normal and hyperplastic lobules, but also poorly developed tubular type lobules and lobules containing cysts. This led finally to a complete disappearance of the fat tissue originally present. The shape and density of the fibroblast nuclei, as well as the thickness and staining qualities of the collagen fibres, helped in deciding the time sequence of proliferative changes. The second pattern showed fibroadenomatous formation either from or in association with an adenoma (Fig. 9). In larger fibroadenomas remnants of previous adenomas could be discerned.

Twenty-three adenomas were found. These invariably occurred in regions of the breast showing marked lobular development and lobular hyperplasia. Adenomas appeared as large circumscribed masses surrounded and partitioned by collagenous tissue (Fig. 10). Cystic change and intra-acinous fibrous papillary projections covered by a single layer of epithelial cells were found in some tumours. It was assumed that adenomas were formed from pre-existing hyperplastic lobules because only in breasts with good alveolar development were these tumours found. In fact it was sometimes difficult to distinguish between an area of gross alveolar hyperplasia and an adenoma. Secretory activity was noted in both these structures ; a feature never seen in the early adenocarcinomas but present in parts of advanced carcinomas.

*Mast cells and myoepithelial cells*

No mast cell reaction was found in the early end-bud and ductal carcinomas or in relation to the early fibroadenomas. However, in tumours of palpable size, an unevenly distributed mast cell reaction was sometimes seen in the stroma around and between the neoplastic epithelial cell masses.

Both the adenocarcinomas and fibroadenomas encountered in this study failed to show a distinct double population of neoplastic cells which might have suggested the presence of a myoepithelial cell contribution to these tumours. Attempts to visualize these elements by means of the alkaline phosphatase activity (Gomori) failed to give evidence of their presence in early tumours. Although staining reactions were obtained in endothelial cells of capillaries, very little staining occurred in healthy non-secretory tumour cells. Equally, Masson's Trichrome method, Mallory's PTAH and H. & E. staining also were of little assistance.

*Evidence of acute changes following the administration of carcinogens?*

Attempts were made to elucidate whether or not changes were present in breast glands of rats treated with DMBA corresponding to those which are so obvious in the skin following application of the carcinogen. Breast glands of rats killed at 48 and 110 hours after receiving 15–17 mg. DMBA failed to reveal with the light microscope any alteration whatsoever and could not be distinguished from the breast glands of untreated virgin animals.

*Breast gland architectural changes associated with ageing and carcinogen administration*

Whereas the early neoplastic lesions gave no hint whether 2-AAF, DMBA or MC had been the carcinogen used to induce them, there were morphological changes in the breast glands in which these tumours appeared suggestive of the agent used. Breast glands of 2-AAF and MC treated rats were indistinguishable from untreated females of the same ages. DMBA treated rats, however, showed an overall lobular development exceeding that seen in the control virgins. Mammary carcinomas were usually found in younger rats lacking the degree of lobular development seen in older animals. Adenomas and fibroadenomas, by way of contrast, were found in older animals possessing well-developed lobules and lobular hyperplasia.

Examination of breat tissues in control rats of various ages demonstrated that with increasing age the breast gland lost its uniform appearance. In 2-months-old Wistar rats of our colony end-buds predominated and lobules were comparatively inconspicuous (Fig. 3 and 5). In older females (aged 25–30 weeks) this uniformity of breast gland appearance was largely lost because of the irregular lobular development and hyperplasia. Although in some animals or in a neighbouring part of the breast in the same animal end-buds and lateral-buds still had a juvenile appearance, most of these structures had either been transformed into well-developed lobules or appeared as blindly-ending hollow sacs. The above changes appeared to progress with age in both degree and frequency—at least up to the age of 48 weeks, the maximum age studied.

### DISCUSSION

The discovery of numerous papillary and non-papillary duct or ductule carcinomas in the rat agrees with the findings of Geschickter (1943), and Nelson (1944), who used oestrogens, and Cantarow, Stasney and Paschkis (1948), Shay, Harris and Gruenstein (1952), Ross *et al.* (1953), Howell (1959) and Huggins, Briziarelli and Sutton (1959) who worked with chemical carcinogens. Mammary adenocarcinomas also arose independently in end-buds. The finding of three purely end-bud carcinomas and thirty combined lesions in which the end-bud and duct changes were often separate entities established the end-bud as a primary centre of neoplastic proliferation. The relative preponderance of duct and ductule lesions may in part be a reflection of the fact that the end-bud, lacking a stout supporting wall of connective tissue, was more rapidly expanded by tumour growth and soon rendered unsafe for interpretation. Huggins and his group observed that in virgin rats treated with chemical carcinogens, carcinomas also frequently arose in acini. Granted that the term acinar was used by Huggins to refer to the structures called end-bud in this present work, then my findings are in agreement with those of the Huggins' group. With one possible exception the lobular carcinomas described by Geschickter (1943) were absent from my material.

No lesions were encountered in the numerous ducts and end-buds examined which could be regarded as precursors of neoplastic transformation. This failure to find pre-neoplastic lesions in the rat was also reported by Scholler and Carnes (1958). Carcinomatous change, apart from one possible exception, did not occur in well-developed or hyperplastic lobules and only one example of carcinoma arising from an adenoma was encountered. Scarf, Ross and Skoryna (1952) found that nipple ligation did not change the final tumour incidence. In the small experiment described here in which unilateral nipplectomies were performed, no significant differences in either tumour incidence or time of tumour appearance were apparent.

In the early fibroadenomatous lesions studied, tumour formation in some instances involved fibroadenomatous change either from or in association with an adenoma. Geschickter (1943) described fibroadenomas containing adenoid proliferations, and Bagg and Hagopian (1939) noted that some fibroadenomas seemed to arise in adenomas. The spontaneous lesions studied by Wright, Klinck and Wolfe (1940) formed a continuous series ranging from adenomas to fibromas.

The benign epithelial lesions especially the adenomas arose in well stimulated breast glands.

Huggins and Yang (1962) defined a number of " critical factors ", including strain, hormonal status, dosage, nature of the agent and age, which influenced the mammary response of animals treated with chemical carcinogens. In the experiments described here fibroadenomas and adenomas showed a definite trend to develop in older animals in association with well developed lobules while carcinomas arose generally in younger animals from ducts and end-buds, i.e tumours arose at a time before all the end-bud regions became converted into ductules and lobules. This suggested a relationship between alveolar development and tumour formation. Huggins and his collaborators and Shay, Gruenstein and Kessler (1962) reported a reduction in mammary cancer incidence if, after the chemical carcinogen was administered, lobular hyperplasia were induced. It has also been well established that the response of rats to chemical carcinogens decreases with age (Bielschowsky, 1947 ; Huggins *et al.*, 1961) and this is well-pronounced when treatment starts at the age of 6 months. As far as it is known, there is no marked difference in the endocrine status of virgin rats at 2 months when the animals are highly susceptible and at 6 months when the animals are much less susceptible. Thus it seems possible that, provided the genetically determined susceptibility is high, then the numbers of undeveloped end-buds may play a role in determining the response to the carcinogen. Once a group of cells has reached a stage in differentiation and functional activity they no longer have the same capacity to replicate and be modified by a carcinogenic agent (Lasfargues and Murray, 1964).

As mentioned previously the breast glands of rats treated with DMBA appeared better developed than those of the controls, and that DMBA treated rats developed fibroadenomatous tumour, rather than carcinomas. Other workers obtained mainly mammary adenocarcinomas when DMBA was used (Huggins *et al.*, 1961 ; Howell, 1959). Geyer *et al.* (1951) and Geyer *et al.* (1953) found mainly adenocarcinomas when high doses of DMBA were given but when lower doses were employed proportionately more fibroadenomas resulted. The dose chosen in this work was probably on the low side for the rats used. Sydnor *et al.* (1962) have shown that the apparent resistance of certain strains to DMBA can be overcome by increased dosage. But even if this is conceded there still remains the question whether the hormone-mimetic action of DMBA as postulated by Jull (1956) can be equated with its tumorigenic activity. As far as the fibroadenomata induced by DMBA are concerned, our findings support Jull's hypothesis but in the case of the carcinomas induced by MC the correlation breaks down. Hormone-mimetic changes were neither seen in this group, nor in the rats treated with 2-AAF.

### SUMMARY

Mammary tumours were induced in virgin rats by 2-AAF, DMBA and MC. Early neoplastic lesions for histological study were discovered by examining appropriately stained and cleared breast glands with the aid of the dissecting microscope.

Mammary carcinoma in the rat is multifocal in origin and tumours arise almost exclusively in ducts, ductules and end-buds without the intervention of any recognizable lesion preceding the appearance of tumour eclls.

In contrast to the carcinomas, fibroadenomas and adenomas arise in older animals with generally well-developed breast glands.

It is suggested that for successful tumour induction the architectural state of the mammary gland is of importance, as well as the action of hormonal factors, genetically-determined susceptibility, the agent and its dosage.

No significant myoepithelial cell contribution was found in the early carcinomas and fibroadenomas and mast cells did not appear to play an essential role in tumour formation.

I would like to acknowledge my indebtedness to the late Dr. F. Bielschowsky for his unfailing encouragement and advice, and also to Dr. Marianne Bielschowsky for her assistance in the management of the experimental animals.

### REFERENCES

Bagg, H. J. and Hagopian, F.—(1939) *Am. J. Cancer*, **35**, 175.
Bielschowsky, F.—(1947) *Br. med. Bull.*, **4**, 382.
Bonser, G. M.—(1945) *J. Path. Bact.* **57**, 413.—(1954) *Ibid.*, **68**, 531.
Bonser, G. M., Dossett, J. A. and Jull, J. W.—(1961) 'Human and Experimental Breast Cancer'. London (Pitman Medical).
Cantarow, A., Stasney, J. and Paschkis, K. E.—(1948) *Cancer Res.*, **8**, 412.
Cheatle, G. L. and Cutler, M.—(1931) 'Tumours of the Breast'. London (Edward Arnold).
Geschickter, C. F.—(1943) 'Diseases of the Breast'. Philadelphia. (J. B. Lippincott).
Geyer, R. P., Bleisch, V. R., Bryant, J. E., Robbins, A. N., Saslaw, I. M. and Stare, F. J.—(1951) *Cancer Res.*, **11**, 474.
Geyer, R. P., Bryant, J. E., Bleisch, V. R., Peirce, E. M. and Stare, F. J.—(1953) *Ibid.*, **13**, 503.
Howell, J. S.—(1959) *Acta. Un. int. Cancr.*, **15**, 163.
Huggins, C., Briziarelli, G. and Sutton, H.—(1959) *J. exp. Med.*, **109**, 25.
Huggins, C.—(1960) 'Biological Activities of Steroids in Relation to Cancer. I. Introduction'. New York (Academic Press), pp. 1–8.
Huggins, C., Grand, L. C. and Brillantes, F. P.—(1961) *Nature, Lond.*, **189**, 204.
Huggins, C. and Yang, N. C.—(1962) *Science*, **137**, 257.
Jull, J. W.—(1956) *Acta Un. int. Cancr.*, **12**, 653.
Jull, J. W. and Huggins, C.—(1960) *Nature, Lond.*, **188**, 73.
Lasfargues, E. Y. and Murray, M. R.—(1964) *Ibid.*, **204**, 593.
Muir, R.—(1941) *J. Path. Bact.*, **52**, 155.
Nelson, W. O.—(1944) *Yale J. Biol. Med.*, **17**, 217.
Noble, R. L. and Cutts, J. H.—(1959) *Cancer Res.*, **19**, 1125.
Ross, R. C., Scarf, R. F. and Skoryna, S. C.—(1953) *Archs Path.*, **55**, 173.
Scarf, R. F., Ross, R. C. and Skoryna, S. C.—(1952) *Surg. Forum. Washington*, (W. B. Saunders), pp. 689–93.
Scholler, J. and Carnes, R. E.—(1958) *Proc. Am. Ass. Cancer Res.*, **2**, 343.
Shay, H., Gruenstein, M. and Kessler, W. B.—(1962) 'The Morphological Precursors of Cancer'. Edited by L. Severi, Perugia. p. 305.
Shay, H., Harris, C. and Gruenstein, M.—(1952) *J. natn. Cancer Inst.*, **13**, 307.
Sydnor, K. L., Butenandt, O., Brillantes, F. P. and Huggins, C.—(1962) *Ibid.*, **29**, 805.
Wright, A. W., Klinck, G. H. and Wolfe, J. M.—(1940) *Am. J. Path.*, **16**, 817.
Young, S., Cowan, Dorothea, M. and Sutherland, Lucy E.—(1963) *J. Path. Bact.*, **85**, 331.